# A novel prognostic signature and potential therapeutic drugs based on tumor immune microenvironment characterization in breast cancer

**DOI:** 10.1016/j.heliyon.2023.e20798

**Published:** 2023-10-07

**Authors:** Yan Zhang, Mingrui Zhou, Jie Sun

**Affiliations:** a Breast Disease Diagnosis and Treatment Center, Central Hospital Affiliated to Shandong First Medical University, Jinan, PR China; b Gastrointestinal Surgery Department I, Shandong Provincial Third Hospital, Jinan, PR China

**Keywords:** Breast cancer, Immune landscape, Potential drugs, Prognostic signature, Tumor microenvironment

## Abstract

Tumor microenvironment (TME) is closely correlated to the occurrence and progression of breast cancer, however its potentiality in assisting diagnosis and therapeutic decision remains unclear. Therefore, the major aim of this study is to explore the prognostic value of TME related gene in breast cancer. Expression matrices and clinical data of breast cancer obtained from public databases were divided into TME relevant clusters according to immune characterization. A 12-gene molecular classifier was generated through the utilization of differentially expressed genes identified between distinct Tumor Microenvironment (TME) clusters, coupled with correlative regression analysis. The performance of this TME-driven prognostic signature (TPS) were examined across both the training and validation cohorts. Furthermore, our study revealed that breast cancer cases classified as high-risk based on the TPS exhibited the phenotype with elevated immune cell infiltration, higher tumor mutational burden, and a notably worse overall prognostic outcome. To conclude, the novel TME-based TPS was able to serve as a superior prognosis indicator for breast cancer, alone or jointly with other clinical factors. Also, breast cancer patients belong to different risk subgroups of TPS were found potentially suitable for distinguished therapeutic agents, which might improve personalized treatment for breast cancer in the future.

## 1 Introduction

Breast cancer (BC) is the most common malignancy worldwide and is also regarded as one major cause of cancer-related death. According to global data, BC accounts for approximately 2,261,419 new cases and 684,996 new deaths in 2020 [1]. As for women, BC represents 30 % of cancers and 15 % of mortality-to-incidence ratio. Despite the vast number of BC patient every year, the way how it is viewed has changes dramatically during the last decades [2]. At the moment, the diagnosis and treatment of BC largely depends on extensively characterized molecular hallmarks such as ER, HER2, Ki-67 and so forth. Based on biomarker combinations, BC is now divided into four subtypes including luminal A, luminal B, HER2 overexpression and triple negative, representing 50 %, 14 %, 13 %, 23 % of total BC patients, respectively. While primary surgery remains the first option of treatment, radiotherapy, chemotherapy, endocrinotherapy, target therapy and immunotherapy have witnessed huge development in the last decades. However, the prognosis of BC is still unoptimistic, particularly in triple negative cases with 5-year overall survival (OS) rate from 23.4 % to 50 % [3]. Therefore, the importance of developing new molecular tools to improve early diagnosis and personalized treatment of BC has been emphasized.

In recent years, there has been accumulating evidence elucidating the pivotal role of the TME in the initiation and progression of a diverse range of malignancies, such as BC, gastric cancer, liver cancer and pancreatic cancer [4]. TME refers to a biological environment in tumor, consisting of malignant cells, non-malignant cells, non-parenchymal cells such as immune cells, stromal cells and biological factors secreted or recruited by these cells [5,6]. Traditional knowledge considers BC as a poorly immunogenic cancer due to its relatively low mutation burden. Nevertheless, a great number of recent studies have drawn contradictory conclusion. Compared with other malignancies, BC is one with remarkable heterogeneity where each subtype shows distinct immunogenic potentiality. Generally, immune infiltration is found most predominant in triple negative BC followed by HER2 positive BC, while luminal B and luminal A type, ER-positive BC found little immune activities [7,8]. In HER2 positive breast cancer, increased tumor-infiltrating lymphocyte (TIL) was found associated with better prognosis [9,10]. Similarly in triple negative breast cancer (TNBC), lymphocyte predominance reflects decreasing risk of distant recurrence [11]. Despite the clinical significance of TME cells and signals are increasingly aware in BC, the capability of TME related genes as molecular biomarker in diagnosis and prognosis of BC is still not clear.

Therefore, in this study we comprehensively evaluate the clinical significance of TME associated genes by analyzing the immune character of BC cohorts obtained from the public database. After the cohort was divided via different immune pattern into main sub-clusters, the TME based prognostic signature (TPS) was generated according to the machine learning algorithm with TME related genes. Then, the cohort was then separated into risk sub-groups according the risk score endowed by TPS. Subsequently, the study thoroughly evaluated the prognostic capabilities of TPS and proposed therapeutic interventions upon the TPS risk profiles. In conclusion, the TME-associated TPS holds significant promise as a valuable tool for diagnosis and therapeutic decision-making process in a large BC population.

## 2 Materials and methods

### 2.1 BC cohort acquisition

The public databases were used to retrieve the RNA transcriptome data and corresponding clinical information of BC patients. The BC cohort of The Cancer Genome Atlas (TCGA-BRCA) including 1109 BCE patients and 113 normal breast tissue was obtained from the TCGA CDC data portal (http://portal.gdc.cacner.gov/repository) and Cbioportal (http://cbioportal.org). For data sets, fragments per million reads (FPKM) and transcripts per million mapped reads (TPM) normalized value was used for further analysis.

### 2.2 Immune landscape in BC

The immune profile of BC was systematically evaluated using the CIBERSORT algorithm, which incorporates the phenotypic data of 22 key immune cell types. CIBERSORT is a deconvolution algorithm that employs a defined set of gene expression markers to estimate the relative proportions of distinct cell types within tumor specimens composed of heterogeneous cellular clusters. The reference gene expression signature for these 22 immune cell types, LM22, was acquired from the CIBERSORT database (http://cibersort.stanford.edu/).

### 2.3 Consensus clustering based on TME profilling

Hierarchical agglomerative clustering method (Euclidean distance and Ward's linkage method) was employed to cluster BC specimens exhibiting similar tumor microenvironment (TME) characteristics. Then based on CIBERSORT-derived immune cell expression profiles, consensus clustering was carried out using the cumulative distribution function (CDF) ranging from 2 to 9 with the R package "ConsensusClusterPlus." Subsequently, differentially expressed genes (DEGs) were identified between these TME clusters using the "limma" R package, employing an threshold of adjusted P value < 0.05.

### 2.4 Establishment of TPS in BC

As the TME related genes was screened out, potential prognostic genes were further identified via the univariate Cox regression with a prerequisite of significant correlations with overall survival (P < 0.05). Next, a random survival forest (RSF) regression model was administrated to eventually generate TPS classifier with the prognostic candidates. As a well-established machine learning model, RSF is considered stable dealing with very high dimensional parameter spaces and reach satisfying predictive efficacy [12,13]. The RSF underwent 1000 iterations and the TPS component genes were selected under the largest C-index value to eventually establish TPS. Afterwards, an TPS risk score was calculated according to the following equation of the TME molecular signature:

$$RiskScore=\sum_{k=1}^{n}Coef_{k}\times {\mathrm{Exp}}_{k}$$

"Coefk" represents gene coefficients, and "Expk" represents gene expression in each sample. Validation cohort samples were given TPS scores and divided into high-risk and low-risk groups based on the mean TPS value. The TME molecular classifier's performance was assessed in both cohorts (training and validation) using Kaplan-Meier analysis, time-dependent ROC curves, multivariate Cox regression, and nomograms.

### 2.5 Functional enrichment analysis among distinct TPS risk groups

To explore potential differences in biological entities between TPS subgroups, functional enrichment analysis was accessed with the R package "clusterProfiler." The identification of differentially expressed genes (DEGs) between distinct TPS risk groups followed the same criteria as applied to TME-related genes (adjusted P < 0.05). Gene set signatures were obtained from the MSigDB database, and Gene Set Enrichment Analysis (GSEA) was utilized to highlight the most significantly changed signaling pathways, biological processes, and gene hallmarks between TPS risk subgroups. Similarly, single-sample gene set enrichment analysis (ssGSEA) compared immune characteristics between these risk subgroups.

### 2.6 Assessment of tumor stromal and immune dysfunction between different risk groups

Immune characteristics, including tumor stromal content and immune dysfunction, were subsequently examined to assess their potential implications for immunotherapy. Stromal scores, immune scores, ESTIMATE scores, and tumor purity were calculated using the "ESTIMATE" R package. Furthermore, Tumor Immune Dysfunction and Exclusion (TIDE) scoring was employed to evaluate immunotherapy response, immune dysfunction, immune exclusion, and microsatellite instability (MSI). Additionally, gene mutation status and tumor mutation burden (TMB) were assessed in the high- and low-risk TPS subgroups.

### 2.7 The identification of potential therapeutic agents targeting TPS risk subgroups

Estimating the drug efficacy through the screening of half-maximal inhibitory concentration (IC50) values in specific TPS-risk subgroups. These drug sensitivity data on human cancer cell lines were derived from publicly available repositories: the Cancer Therapeutics Response Portal at the Broad Institute (CTRP, http://portals.broadinstitute.org/ctrp) and the Genomics of Drug Sensitivity in Cancer at the Sanger Institute (GDSC, http://www.cancerRxgene.org). Then the large-scale gene expression and drug screening datasets were utilized as training sets to construct ridge regression models using R package "oncoPredict" to predict drug sensitivity in new gene expression datasets.

### 2.8 Statistical analysis

All statistical analyses were conducted using the R software platform (v4.1.0, R Foundation for Statistical Computing, Vienna, Austria). Key R packages utilized included "limma," "survival," "ROCR," "ggplot2," and "caret."

For the comparison of variables across multiple groups, parametric factors were assessed using Student's t-test and ANOVA analysis, while nonparametric factors were evaluated using the Wilcoxon rank-sum test and Kruskal-Wallis test.

To measure the correlation between different variables, both Spearman's rank-order correlation and Pearson's r correlation were employed. Survival analysis was conducted using Kaplan-Meier analysis along with the log-rank test. To assess the efficacy of receiver operating characteristic curves, the area under the curve (AUC) was calculated. In all statistical analyses, significance was determined by a two-tailed P-value of less than 0.05.

## 3 Results

### 3.1 TME landscape of BC

**Fig. 1:**
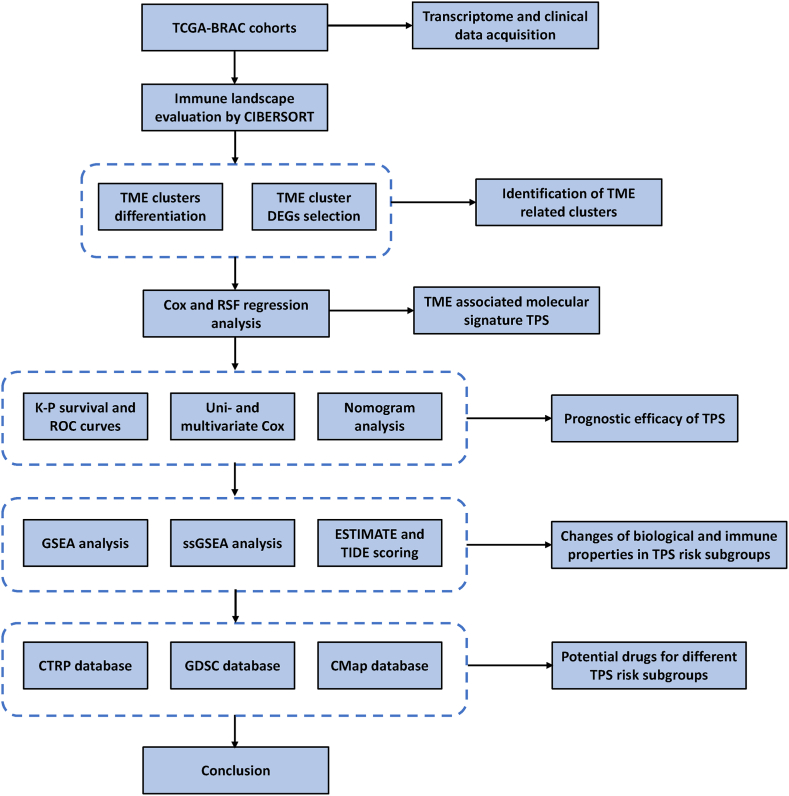
Scheme of the study.

To emphasize, the TME landscape of BC was thoroughly evaluated and establish a TME associated prognostic signature to assist diagnosis and customized treatment of BC. In specific, the scheme of this work was shown as follow (Fig. 1).

**Fig. 2:**
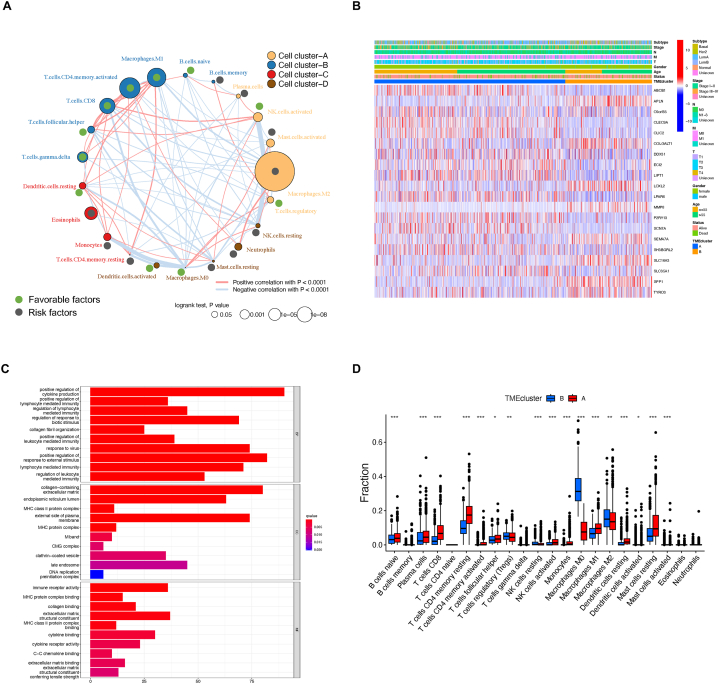
The characteristics of TME clusters in BC. (A) Cellular interactions among TME risk subtypes were displayed with color-coded cell types. (B) Top DEGs between TME related cluster combing with annotation of major clinicopathological factors in TCGA-BRCA cohort. (C) Bar plot illustrating biological changes observed between TME clusters. (D) Alterations in immune cell infiltration between TME clusters (CIBERSORT score). Asterisk symbols reflect significant differences among groups. One asterisk (*), two asterisks (**), three asterisks (***) represents the *P* value less than 0.05, 0.01 and 0.001, respectively. (For interpretation of the references to color in this figure legend, the reader is referred to the Web version of this article.)

CIBERSORT scoring was applied to summarize the TME pattern of each patient in the TCGA-BRCA cohort. The TME immune network was subsequently constructed using CIBERSORT scores to measure cell clustering, cell interactions, and their correlation with the survival of BC patients (Fig. 2A). Colors of node represent distinguished clusters of immune cells, while the size of each node reflects its survival impact. Favorable cell types are filled with green dots while risk cells with grey dots. The thickness of lines indicates interactions between cell types estimated via Spearman correlation. Noticeably, Macrophage M1 and activated CD4 T cells were the most favorable cell types for the OS of BC patients, while Macrophage M2 were the significant risk factor. Notably, a significant correlation was detected between NK cells and macrophages, hinting at the possibility of these two cell types synergistically cooperate in the process of BC initiation and development.

Then, cluster analysis was performed to test if the BC cohort can be grouped according to similar immune background. Unsupervised hierarchical clustering was executed using CDF range of 2–9 to identify the optimal number of clusters. The data revealed that the TCGA cohort could be optimally segregated into two clearly distinct clusters based on TME cell infiltration (Supplementary Figs. 1A–C). Subsequently, 1878 TME related genes were identified between the clusters. The correlation between the TME clusters and the major pathological factors as well as the most significant TME related genes were shown via heatmap (Fig. 2B).

Next, Gene Ontology (GO) functional analysis was applied to investigate the possible difference in biological activities between TME clusters using DEGs (Fig. 2C). To date, positive regulation of cytokine production, positive regulation of lymphocyte mediated immunity, and regulation of lymphocyte mediated immunity were most enriched biological processes; Collagen-containing extracellular matrix, endoplasmic reticulum lumen and MHC class II protein complex were most aggregated cellular component; Immune receptor activity, MHC protein complex binding and collagen binding were most enriched molecular function.

In parallel, activities of major immune cells were also compared between TME clusters via CIBERSORT (Fig. 2D). As expected, significant differences were observed in immune cell infiltration when comparing to the TME clusters. The cluster A exhibited elevated multiple B cells, T cells, NK cells, and Macrophage M1 type, while cluster B patients, in particular, showed higher levels of Macrophage M0 and M2.

### 3.2 Establishment of TPS in BC

The cohort of 1095 TCGA-BRCA patients was segregated into two groups: TME cluster A, comprising 751 patients, and cluster B, encompassing 344 patients. Subsequently, an initial set of 1878 candidate cluster DEGs were identified as TME-associated genes, following the criteria described earlier. On the other hand, 1747 genes were screened out as prognostic significant genes via univariate Cox regression (*P* < 0.05). Eventually, 258 genes were selected by intersecting the DEGs and prognostic genes for further analysis. A RSF model was constructed based on selecting target genes with minimal depth. The RSF went through 1000 times iterations under the criterion of largest C-index value and eventually led to a 40-mRNA molecular classifier, leading to a 12-gene containing TPS of BC. The genetic information on the component genes was listed as follows (Table 1).

**Table 1:** Genetic detail of the candidate genes in TPS.

Ensembl ID	Symbol	Complete name
ENSG00000102144	PGK1	Phosphoglycerate kinase 1
ENSG00000179363	TMEM31	Transmembrane protein 31
ENSG00000135537	AFG1L	AFG1 like ATPase
ENSG00000186153	WWOX	WW domain containing oxidoreductase
ENSG00000180096	SEPTIN1	Septin 1
ENSG00000114405	C3ORF14	Chromosome 3 open reading frame 14
ENSG00000077585	GPR137B	G protein-coupled receptor 137B
ENSG00000198298	ZNF485	Zinc finger protein 485
ENSG00000111674	ENO2	Enolase 2
ENSG00000196576	PLXNB2	Plexin B2
ENSG00000277758	SYT15B	Synaptotagmin 15B
ENSG00000121058	TBX2	T-box transcription factor 2

## 4 Genes based TPS classifier for BC

Next, the expression of candidate genes was compared in tumor and adjacent normal tissue (Supplementary Fig. 2A-L). Notably, 7 TPS genes were significantly elevated in BC while 3 members decreased. Moreover, the correlation among TPS genes was also investigated via Spearman's correlation analysis (Supplementary Fig. 3A). As a result, the expression of most members failed to show strong correlation with other TPS genes.

**Fig. 3:**
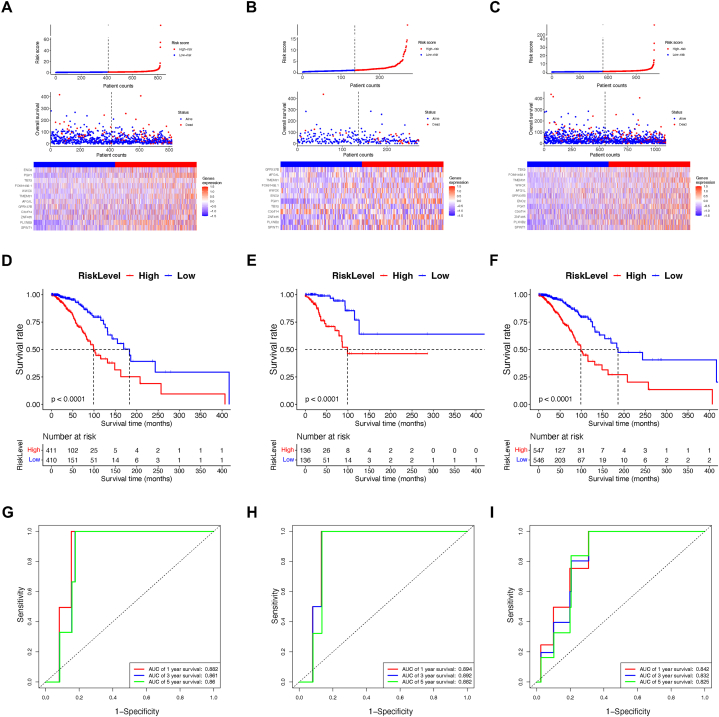
Assessment of TPS as a prognosis indicator. (A) Correlative distribution of TPS risk score and patient counts, vital status and elemental genes in the training group. (B) Correlative distribution of TPS risk score and patient counts, vital status and elemental genes in the testing group. (C) Correlative distribution of TPS risk score and patient counts, vital status and elemental genes in the whole group. (D) Kaplan-Meier survival compared OS in different risk subgroups of the training cohort. (E) Kaplan-Meier survival compared OS in different risk subgroups of the testing cohort. (F) Kaplan-Meier survival compared OS in different risk subgroups of the whole cohort. (G) The ROC of TPS in the training cohort. (H) The ROC of TPS in the testing cohort. (I) The ROC of TPS in the whole cohort.

The risk score, which was generated from the TPS, was computed using the coefficients of the constituent genes, as detailed in previous descriptions. Following this, every sample in both cohorts assigned a risk score and was classified into either a high-risk or low-risk subgroup, with the classification being determined by comparing the sample's risk score to the median value within its corresponding group. The associations between the TPS risk score, survival status, and the expression patterns of TPS genes within these subgroups are detailed as follows (Fig. 3A–C).

### 4.1 Assessment of prognostic efficiency of TPS

TPS score was generated and then its potential as a prognostic indicator was assessed using various methodologies. At first the Kaplan-Meier survival regression analysis was employed to compare OS between high-risk and low-risk groups across both training and validation cohorts. Consistently in all cohorts, the OS of BC patients from high-risk group of TPS was found remarkably shortened compared to their counterparts in the TPS low-risk groups (*P* < 0.001), suggesting TPS was ideal to distinguish CRC patients with distinct outcomes (Fig. 3D–F).

Subsequently, time-dependent receiver operating characteristic (ROC) analysis was conducted to evaluate the prognostic efficacy of TPS in BC. Notably, the AUCs of TPS exhibited high values, indicative of its capacity as a prognostic tool with high sensitivity and specificity.

Specifically, in the training group, the AUCs for TPS were 0.882, 0.861, and 0.860 of OS prediction at 1, 3, and 5 years. In the testing cohort, these values were 0.894, 0.892, and 0.882, and in the combined dataset, they were 0.796, 0.786, and 0.819 (Fig. 3G–I).

In summary, TPS and its scoring system demonstrated remarkable effectiveness in predicting the prognosis of BC, underscoring its significant potential as a novel approach to enhance breast cancer diagnosis and assist in clinical decision-making.

Furthermore, to further evaluate the prognostic potential of TPS, especially with other clinicopathological factors, Cox regression analysis was subsequently conducted. This analysis aimed to investigate the independent prognostic capability of each factor (Supplementary Figs. 4A–B). TPS scoring was assessed with age, TMN, and AJCC pathological stage. After a tow-step test of univariate and multivariate Cox analysis, only the age of patient and the TPS risk score were identified as independent prognostic indicators. The risk score of TPS exhibited significantly correlation (*P* < 0.001). Next, a consensus prognostic model involving TPS and other clinical factors was built with a nomogram. In detail, each factor was assigned a prognostic score based on its clinical significance regarding the outcome. Then the cumulative score reflected the OS probability at 1, 3, and 5 years (Supplementary Fig. 4C). Remarkably, TPS held a predominant position in weights determination, highlighting its pivotal role in the prognostic assessment of the CRC population.

Despite the TPS has exhibited superb prognostic efficacy in predicting OS of BC, it is of great interests to further explore its potentiality in the recurrence of BC. The K-M analysis indicated that the recurrence status of BC patients between high-risk and low-risk group of TPS also significantly differed (P = 0.0011) (Supplementary Fig. 4D). Furthermore, the AUCs of TPS in predicting the recurrence of BC were 0.749, 0.684 and 0.708 for 1, 3 and 5 years (Supplementary Fig. 4E).

### 4.2 Functional analysis of TPS risk subgroups

**Fig. 4:**
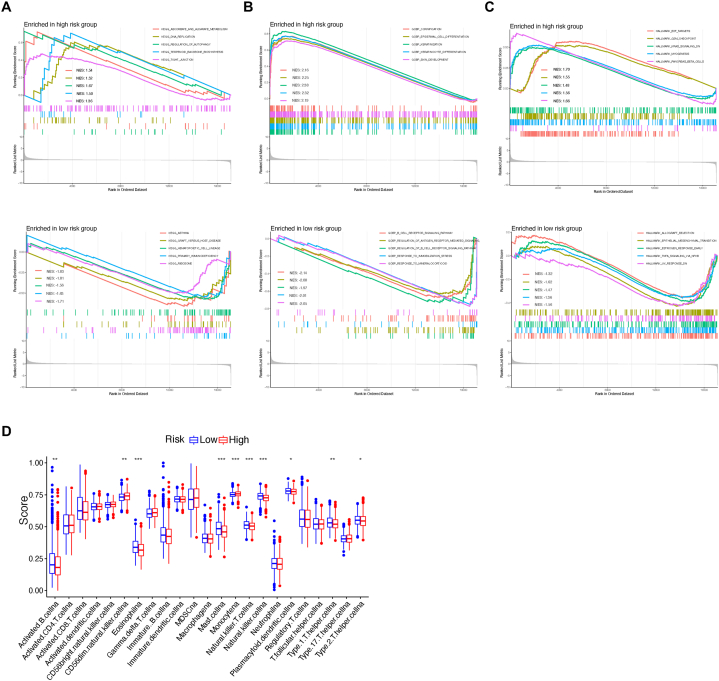
Gene set enrichment shows biological characteristics alterations between TPS risk subgroups. (A) GSEA reveals distinct KEGG signaling pathways. (B) GSEA highlights divergent biological processes. (C) GSEA identifies disparities in hallmark gene sets. (D) The immune landscape is depicted through ssGSEA between groups. Asterisk symbols reflect significant differences among groups. One asterisk (*), two asterisks (**), three asterisks (***) represents the *P* value less than 0.05, 0.01 and 0.001, respectively.

To better understand the possible changes of biological processes in patients from different risk subgroups of TPS, functional analysis was conducted using DEGs between high-risk and low-risk groups. Consequently, we conducted GSEA to identify the top enriched KEGG signaling pathways, GO biological processes (BP), and GO hallmarks. Simultaneously, we examined immune activities through single-sample Gene Set Enrichment Analysis (ssGSEA). As a result, KEGG pathways of ascorbate and aldarate metabolism, DNA replication, regulation of autophagy, were the top enriched pathways in the TPS high-risk group, while asthma, graft host disease, and hematopoietic cell lineage were most aggregated in the low-risk group (Fig. 4A). For GO BP, cornification, epidermal cell differentiation and keratinization were closely correlated to the high-risk group. To compare, B cell receptor signaling, regulation of antigen receptor mediated signaling, and response to immobilization stress were strongly assembled in to low-risk group (Fig. 4B). Hallmark was a group of genes representing same biological activity defined by MSigDB. Hallmarks of E2F targets, G/M checkpoint, KRAS signaling were associated to high-risk group of TPS, and allograft rejection, epithelial mesenchymal transition, estrogen response were successfully enriched in the low-risk group of TPS (Fig. 4C).

Given that TPS serves as a molecular classifier relying on TME characterization, the significant altered immune cell activities between different TPS risk subgroups was measured via ssGSEA (Fig. 4D). To date, infiltration of some critical immune cells was found differential activated between different risk subgroups. NK cell, activated B cells, T helper cell, and mast cell, were found significantly elevated in the low-risk group, while only the monocyte was increased in the high-risk group. The rest level of immune cells remains unchanged.

### 4.3 TME background and tumor mutation status in TPS risk subgroups

**Fig. 5:**
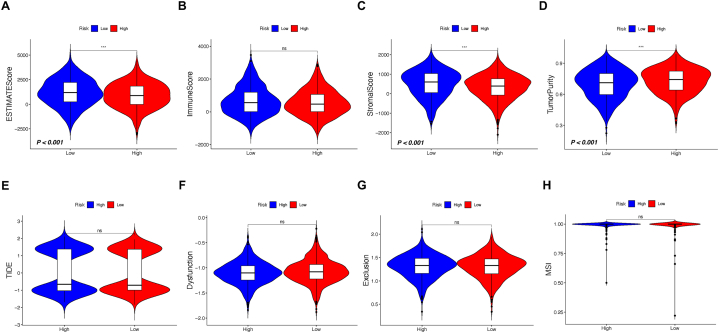
Assessment of tumor purity and potential responsiveness to immunotherapy across TPS-risk subgroups. (A) ESTIMATE score comparison. (B) Immune scores comparison. (C) Stromal scores comparison. (D) Tumor purity comparison analysis. (E) TIDE score comparison. (F) Assessment of immune dysfunction score. (G) Assessment of immune exclusion score. Asterisk symbols reflect significant differences among groups. One asterisk (*), two asterisks (**), three asterisks (***) represents the *P* value less than 0.05, 0.01 and 0.001, respectively.

Immune activity and Tumor Mutation Status in TPS-Risk Subgroups Although TPS originates from clusters with diverse TME backgrounds, the link between the potential impacts of immunotherapy and TPS risk remains uncertain. Consequently, we applied the ESTIMATE algorithm to further anticipate the extent of immune activity in BC, specifically between the high-risk and low-risk groups (Fig. 5A–D). In detail, the level of ESTIMATE score and stromal score of the high-risk group was found inferior to low-risk group, while tumor purity was higher in the high-risk group and immune score remains equal in both risk subgroups. Theses result implies that the TPS might be used as a tool judging the immune infiltration status for the BC population, and down-regulated immune activity might be a sign for worse prognosis and poor reaction to immune checkpoint therapy.

However, the results of TIDE scoring including immune dysfunction and immune exclusion failed to show difference between risk subgroups of TPS, implying that the increased level of immune infiltration might also not guarantee the effect of immunotherapy (Fig. 5E–H).

**Fig. 6:**
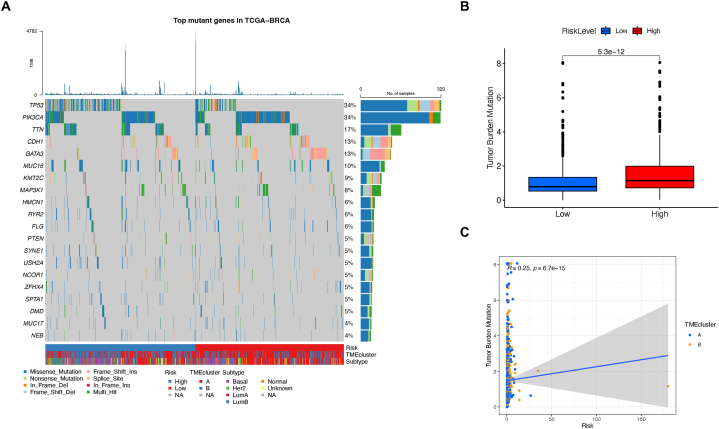
Gene mutation status analysis between TPS risk subgroups. (A) Top somatic mutation genes in TPS risk subgroups. (B) Tumor mutation burden comparison. (C) tumor mutation burden and TPS risk score’ correlation.

Lastly, we explored the potential association between TPS and somatic mutation burden. An oncoPlot was generated to visualize the common mutant genes within the TPS risk subgroups (Fig. 6A). To compare, TP53 and PIK3CA remains the most frequent mutations in the whole BC population. Nonetheless, TP53 was the top mutant gene in the low-risk group of TPS, while PIK3CA was more genetically changed in the high-risk group. Next, we observed a significant variation in tumor mutation burden across different risk categories of TPS (Fig. 6B). Specifically, the degree of tumor mutation burden exhibited a strong correlation with the TPS risk score, as displayed by the Spearman coefficient (*R* = 0.25, P < 0.001, Fig. 6C).

### 4.4 Exploration of potential therapeutic drugs for patients in high-risk group

**Fig. 7:**
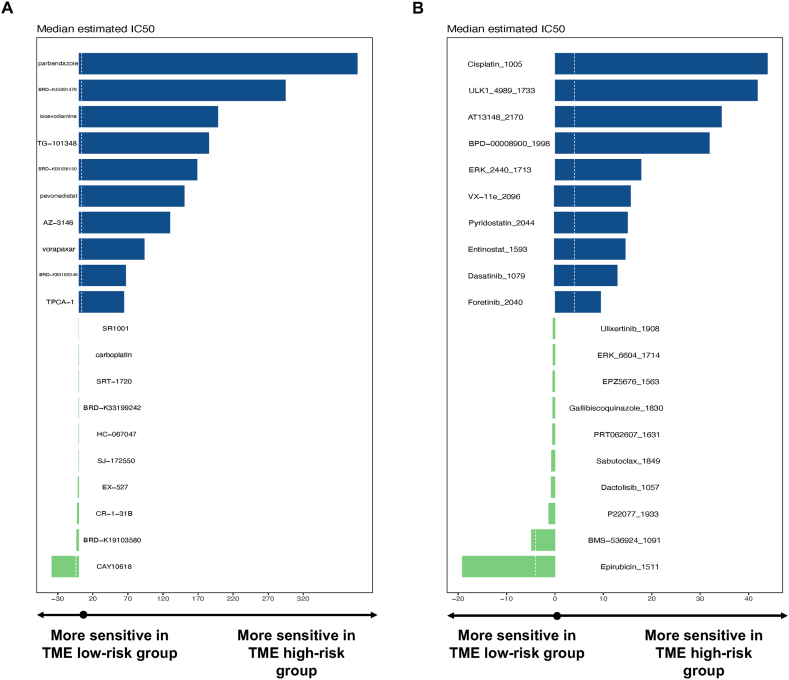
Identification of Potential Drug Candidates Tailored for Distinct TPS Risk Patient Subgroups. (A) Candidate drugs identified within the CTRP database. (B) Candidate drugs identified within the GDSC database.

According to the results above, BC patients with different TPS score showed distinct biological pattern and TME character, implying personalized treatment based on TPS grouping might be beneficial for some group of BC patients. Hence, we searched for drugs specific to the TPS risk subgroup within the extensive databases CTRP and GDSC, which encompass drug sensitivity data for a diverse range of novel drugs and small molecules, as previously mentioned. Then Wilcoxon analysis was applied to measure the correlation between the risk score and the median IC50 values of all compounds. As a result, ratio of normalized IC_50_ value between the low- and high-risk groups was compared to acquire the most sensitive drugs for patients from different risk subgroups, respectively. Top 10 drugs for each risk subgroup were listed as followed (Fig. 7A–B). In the CTRP database, parbendazole, BRD-K45681478, and isoevodiamine were identified as the most effective agents for high-risk patients, whereas CAY10618, BRD-K19103580, and CR-1-31B were specialized for low-risk patients. In the GDSC database, high-risk patients were found to benefit from cisplatin, ULK1-4989, and AT3148, while low-risk patients of TPS were associated with epirubicin, BMS-536924, and P22077 as potential therapeutic options.

Aside from databases of CTRP and GDSC, the CMap mode-of-action (MoA) database including nearly 3000 small-molecule compounds was also administrated. The CMap algorithm compares the expression profile of mRNA in different TPS risk subgroups with the response pattern of drugs or genetic changes in the library. Generally, candidate drug with a CMap score of less than −95 would be considered significantly effective. Thus, Top drugs from CTRP and GDSC, as well as representative molecules with low CMap score were listed below (Table 2).

**Table 2:** Information of candidate agents identified by CTRP, GDSC and CMap databases.

Name	Description	CMap score
Parbendazole	Tubulin inhibitor	10.97
TG-101348	FLT3 inhibitor	−73.66
Entinostat	HDAC inhibitor	−94.1
BIX-01338	Histone lysine methyltransferase inhibitor	−99.44
PIK-90	PI3K inhibitor	−99.12
KU-0063794	MTOR inhibitor	−99.12

### 4.5 Candidate molecules identified by public databases

.

## 5 Discussion

BC remains the most common malignancy worldwide and most fatal tumor for women population. The incidence varies among nations and regions but also witnesses an increased tendency toward younger age, possibly a result of fast popularization of mammography. Moreover, due to high quality prevention, early detection, and improved therapeutic services, the mortality of the disease has been continued to decline during the last decades. The overall death rate of BC has declined by 43 % from 1975 to 2020 in the US [14]. However, the prognosis of patients with metastatic BC is still poor, and the mechanisms of tumorigenesis as well as the progression of the disease remains obscure. Therefore, it is substantial to develop novel biomarkers to assist diagnosis and treatment of BC in the future.

As a result, the number of molecular signatures associated to the prognosis of BC has grown rapidly over the last years. For instance, Su et al. has developed a 19-gene molecular signature to predict the overall survival of BC [15]. Other groups have also built prognostic classifiers based on genes related to diverse biological aspects of BC including ferroptosis, cuproptosis, oxidative stress and methylation changes [[16], [17], [18], [19]].

Meanwhile, growing evidence has gradually demonstrated the critical role of immune system in all etiological stages of BC including tumor growth, metastasis, angiogenesis, recurrence and response to therapy [20]. In the early age, BC was considered only with little immune involvement, largely due to low mutation rate. However, the presence of immune cells in the microenvironment of certain subtypes of BC, especially in the patients of triple negative and HER2-overexpression has been gradually emphasized. Noticeably, increasing number of literatures have demonstrated that the immune cells in the TME were closely correlated to tumor growth and progression of BC, and therefore were ideal to serve as prognostic indicator and therapeutic targets for BC. The major types of immune cells contained in the TME of BC are T lymphocytes, B lymphocytes, macrophages, NK cells and dendritic cells, and the complex of immune infiltration can either establish a protective antitumor response or conversely induce chronic inflammation that promotes disease progression [[21], [22], [23]]. Despite the potential of immune cells in the BC as prognostic indicator has been increasingly recognized, and a number of immunogenetic classifiers for certain subtypes of the disease have been developed, novel biomarkers with high accuracy and specificity and of clinical importance are still in great need.

In this study, the TME based prognostic signature TPS for BC was constructed and thoroughly evaluated. To date, the TCGA-BRCA cohort was accessed and employed as the major training data set. The cohort was stratified into two distinct TME clusters based on the activity levels of primary immune cells, as determined by CIBERSORT scoring. These clusters exhibited notable differences in biological characteristics, with a particular focus on immune features. In addition, DEGs of the clusters were recognized as TME related genes and selected to build the prognostic classifier. Instead of methods commonly used such as LASSO or RIDGE regression, the study applied random survival forest to achieve high prognostic efficacy in the novel TME associated classifier TPS.

As a result, the TPS split the cohort of BC into a high-risk and a low-risk subgroup via the median value of the risk score based on the expression of TPS component genes in each patient. Notably, in both the training and validation cohorts, high-risk patients, as defined by TPS, displayed significantly reduced OS compared to their low-risk counterparts, implying that TPS was ideal for separating patients with different risk and survival expectancy.

Additionally, the ROC test reflecting prognostic sensitivity and specificity was also used. Intriguingly, The TPS showed superb capacity of prognostic indication, where its AUCs reached 0.88, 0.86 and 0.86 in 1-, 3-, and 5-year survival anticipation in the training cohort. Similarly, the AUCs achieved as high as 0.89, 0.89 and 0.88 for 1-, 3-, and 5-year survival in the testing group and 0.80, 0.79 and 0.82 for 1-, 3-, and 5-year survival in the whole group of TCGA BC cohort.

Altogether, our result shows TPS is a stable, robust and effective prognostic indicator for BC that not only suffice to be an independent prognostic factor, but also can well coordinate with other clinical factors to improved prognostic efficacy.

Therefore, TPS was composed of 12 genes that probably correlate to the occurrence and development of BC. Transmembrane protein 31 (TMEM31) was the element gene of TPS with most coefficient value. Recently, it has been recognized as one of the cancer/testis (CT) antigens which normally restricted in the germ cells of the testis in healthy tissue but also surprisingly up-regulated in the malignant tumor of melanoma [24,25]. The expression characteristics of TMEM31 was investigated by comparing the level of TMEM31 in primary melanomas and metastatic ones. Surprisingly, the expression of TMEM31 was found significantly increased in metastatic melanomas compared to in primary tumors. As a newly discovered biomarker, little is known about the biological role and mechanism of TMEM31 in melanoma as well as other malignancies. Other members of CT antigen family such as MAGE-A1, MAGE-A4 and NY-ESO-1 have shown a clear correlation to advanced tumor stage in melanoma [26]. Altogether, TMEM31 might play critical role in the process of tumor metastasis. To our knowledge, CT antigens are of high immunogenicity and therefore often serve as immunotherapeutic target, which reinforces the finding in this study that TPS differs BC of distinct prognosis and expectancy of immunotherapy. However, the specific function and underlying molecular mechanism of how TMEM31 in BC remains largely unknown.

T-box transcription factor 2 (TBX2) belongs to T-box family of transcription factors. Since its discovery, the T-box family received great attention in developmental biology. A series of studies revealed that T-box family members expressed throughout critical processes in development [27,28]. The mutation of T-box genes would lead to various genetic disorders such as ulnar-mammary syndrome and cleft palate with ankyloglossia [29,30]. Noticeably, a number of recent studies unraveled that the potential role of T-box members in manipulating the genesis and progression of cancers including melanoma, small cell lung cancer, liver cancer, pancreatic cancer and BC. Specifically, amplified TBX2 in BC might acts as a mediator of cell senescence and thus promotes tumor progression via repressing tumor suppressors such as p16 or p21 in cell cycle (TBX1). Nonetheless, other possible mechanism of TBX2 in BC is still poorly understood. Despite little is known at the moment, the component genes of TPS might shed new light on future research of BC.

To further explore the mechanism behind TPS, we assumed that the change in biological, in particular immune characters between different risk subgroups might be the key. Our result showed that certain biological patterns that prompt tumor progression were strongly aggregated in the high-risk subgroup of TPS. The KEGG pathway of DNA replication, the biological process of epithelial mesenchymal transition, and the hallmark gene set of KRAS signaling are known to have strong correlation to tumor progression. In parallel, immunogenic ssGSEA exhibited that the infiltration of some major immune cells was substantially altered in high-risk group of TPS. Activities of B lymphocyte, NK cell, mast cell and dendritic cell were significantly decreased in the high-risk group of TPS compared with their peers in the low-risk group.

To sum up, the results provide valuable information that could explain the reason behind the TPS classification of BC. Also, the comparison between the risk subgroups might lead to the conclusion that BC patients from the high-risk group are tend to have lower level of immune activity and thus less effective with immunotherapy. However, clinical study of large scale will be needed in the future to further assess the clinical value of TPS in BC.

Since TPS differs BC patients with disparate biological and immunological properties, therapeutic agents possibly specialized for certain risk group were also searched in public drug databases, in particular for high-risk patients of TPS with worse prognosis. To date, top 10 drugs of most effective for each risk subgroup were listed from the CTRP and GDSC as described above, respectively. According to the result from CTRP database, Parbendazole was the most effective drug for TPS high-risk patients. Parbendazole is a potent inhibitor of microtubule assembly by destabilizing tubulin [31]. Parbendazole has been widely used as a major anthelmintic in livestock industry for more than half a century [32,33]. Similar to our finding, a number of studies also discovered Parbendazole as possible anti-tumor agent against pancreatic cancer and head and neck squamous cell carcinoma (HNSCC) [[34], [35], [36]]. However, no experimental or clinical study have been done.

Isoevodiamine, also known as Evodiamine, is a chemical compound extracted from the fruit *Evodiae fructus* [37]. Accumulating evidence has unraveled the anti-tumor effect of Evodiamine both in vitro and in vivo by inhibiting proliferation, repressing metastasis, and inducing apoptosis in various tumor types [[38], [39], [40]]. To note, Evodiamine inhibited the proliferation of BC cell lines in a concentration-dependent manner, while induced apoptosis via up-regulation of caspase 7 activation [41]. Furthermore, Du et al. has also reported that Evodiamine suppressed the ability of metastasis of BC cell by regulating the ERK and p38 MAPK signaling pathway [42].

From database GDSC, Cisplatin, one of the most widely used drug for chemotherapy, was identified as the champion for TPS high-risk patients. Early as 1980s, Cisplatin was used in clinical study of treating metastatic BC and received active result [43,44]. Nowadays, Cisplatin is considered as one of the fundamental drugs in standard chemotherapy for BC, and usually combining applied with endocrinotherapy and immunotherapy to treat advanced BC [44,45].

Additionally, the CMap analysis also carried out a number of novel molecules with strong therapeutic potentiality specialized for high risk patients of TPS. To our concern, drugs that exhibited consistently active effect in multiple databases are more likely to become clinical solution in the future.

TG-101348, also known as Fedratinib, is a selective JAK antagonist that picked concordantly by CTRP and CMap. As a selective JAK antagonist, Fedratinib was extensively studied in hematology both in vitro and i*n vivo*. Moreover, clinical trails are evaluating the role of this promising agent in leukemia and myeloproliferative neoplasm [46]. Yet little is known about the influence of Fedratinib on solid tumors, including BC, and more work are required to be done.

Paradoxically, the therapeutic impact of some molecules in one database disagreed with the result in another. For instance, Parbendazole was identified in CTRP as one of the champions in treating the high-risk group of TPS. However, it was recognized as ineffective in CMap. One possible explanation is that the drug showing insignificant effectiveness for the vast population of BC might be beneficial in treating a small sub-group of patients. Last but not least, the agents raised in this study were substantially more effective for high-risk patients of TPS compared with their counterparts in low-risk group. This observation implies that it is better to genetically examine and group BC patients prior to selecting further treatment of target-, chemo-, endocrino- and immunotherapy.

There are several limitations in this study. First, this study is based on analyzing data from public databases, and further validation of TPS in large-scale cohort is needed in the future. Second, the molecular mechanism of TPS in BC is still unclear and awaits further experiments in the lab.

## 6 Conclusion

To summarize, this study establishes an immunogenic molecular signature to predict the prognosis and improve customized therapy in BC. BC cohorts from public databases were employed and divide into two clusters bsed on their TME character, which followed by the application of RSF model to develop the TPS. Multiple approaches were employed to validate the prognostic efficacy of the TPS classifier. Additionally, the study also examined the differences in biological and immune characteristics between risk subgroups defined by TPS. Finally, potential therapeutic agents were identified for each subgroup. Taking together, TPS might shed new light on the prognosis judgement and personalized treatment of the BC in the future, but more works are needed to be done before the conclusion of this study can be translated into clinical approaches.

## Additional information

The data set used in the study was accessed from the TCGA CDC data portal (http://portal.gdc.cacner.gov/repository) and Cbioportal (http://cbioportal.org). Drug sensitivity data on human cancer cell lines (CCLs) were acquired from Cancer Therapeutics Response Portal (http://portals.broadinstitute.org/ctrp) and Genomics of Drug Sensitivity in Cancer, Sanger institute (http://www.cancerRxgene.org).

## Author contribution statement

Jie Sun conceived and designed the experiments. Yan Zhang and Mingrui Zhou performed the experiments, analyzed and interpreted the data. Yan Zhang wrote the paper. Mingrui Zhou contributed reagents, materials, analysis tools or data.

## Funding information

Not applicable.

## Declaration of competing interest

The authors declare that they have no known competing financial interests or personal relationships that could have appeared to influence the work reported in this paper.
