# Supplementary material for: A novel prognostic signature and potential therapeutic drugs based on tumor immune microenvironment characterization in breast cancer
Source: Heliyon. 2023 Oct 7;9(10):e20798. doi: 10.1016/j.heliyon.2023.e20798 (PMC10582509; doi:10.1016/j.heliyon.2023.e20798)
Supplement: Multimedia component 1 [file mmc1.docx]

**Supplementary Figures**

**Supplementary figure 1. Clustering of TCGA-BRCA based on TME character.** (A) Unsupervised clustering based on TME pattern with CDF value of 2. (B) Unsupervised clustering based on TME pattern with CDF value of 3. (C) Unsupervised clustering based on TME pattern with CDF value of 4.

**Supplementary figure 2. Expression of TPS member genes in TCGA cohort.** (A) Expression of PGK1. (B) Expression of TMEM31. (C) Expression of AFG1L. (D) Expression of WWOX. (E) Expression of SEPTIN1. (F) Expression of C3ORF14. (G) Expression of GPR137B. (H) Expression of ZNF485. (I) Expression of ENO2. (J) Expression of PLXNB2. (K) Expression of SYT15B. (L) Expression of TBX2.

**Supplementary figure 3. Expression relationship among TPS member genes.**

**Supplementary figure 4. Prognostic assessment of TPS combining with clinicopathological factors.** (A) Significant factors in Uni-variate Cox regression. (B) Significant factors in Multi-variate Cox regression. (C) Nomogram including TPS and other clinical factors. (D) Kaplan-Meier survival compared disease free time in different risk subgroups. (E) The ROC of TPS in predicting the recurrence of BC.
